# Neoadjuvant Pertuzumab Plus Trastuzumab in Combination with Docetaxel and Carboplatin in Patients with HER2-Positive Breast Cancer: Real-World Data from the National Institute of Oncology in Poland

**DOI:** 10.3390/cancers14051218

**Published:** 2022-02-26

**Authors:** Agnieszka Irena Jagiełło-Gruszfeld, Magdalena Rosinska, Małgorzata Meluch, Katarzyna Pogoda, Anna Niwinska, Renata Sienkiewicz, Aleksander Grous, Paweł Winter, Zbigniew I. Nowecki

**Affiliations:** 1 Breast Cancer and Reconstructive Surgery Department, Maria Sklodowska-Curie National Research Institute of Oncology, 02-781 Warsaw, Poland; malgorzata.meluch@pib-nio.pl (M.M.); katarzyna.pogoda@pib-nio.pl (K.P.); anna.niwinska@pib-nio.pl (A.N.); renata.sienkiewicz@pib-nio.pl (R.S.); aleksander.grous@pib-nio.pl (A.G.); pawel.winter@pib-nio.pl (P.W.); zbigniew.nowecki@pib-nio.pl (Z.I.N.); 2 Department of Oncological Mathematics, Maria Sklodowska-Curie National Research Institute of Oncology, 02-781 Warsaw, Poland; magdalena.rosinska@pib-nio.pl

**Keywords:** breast cancer, neoadjuvant chemotherapy, elderly, HER2, pathological complete response, safety

## Abstract

**Simple Summary:**

Neoadjuvant chemotherapy in HER2-positive breast cancer has become the standard of care. Systemic treatment allows, in many cases, a reduction in the scope of surgical treatment. The aim of our retrospective study was to confirm the efficacy and safety of neoadjuvant chemotherapy in the TCbH-P program. All study patients received six courses of chemotherapy. All patients achieved a reduction in breast tumour size. There were no cardiac complications in any of the patients that prevented the continuation of treatment. A complete pathological response was achieved in 52.9% of patients. No pCR was achieved in any patient over 60 years of age with luminal B HER2-positive cancer, which questions the use of the TCbH-P regimen in this group of patients.

**Abstract:**

Neoadjuvant systemic therapy has now become the standard in early breast cancer management. Chemotherapy in combination with trastuzumab +/− pertuzumab targeted therapy can improve the rates of pathologic complete response (pCR) in patients with HER2-positive breast cancer. Achieving a pCR is considered a good prognostic factor, in particular, in patients with more aggressive breast cancer subtypes such as TNBC or HER2-positive cancers. Furthermore, most studies demonstrate that chemotherapy in combination with trastuzumab and pertuzumab is well tolerated. The retrospective analysis presented here concentrates on neoadjuvant therapy with the TCbH-P regimen, with a particular emphasis on patients over 60 years of age. We analysed the factors affecting the achievement of pCR and present the adverse effects of the applied therapies, opening discussion about optimizing the therapy of older patients with HER-2 positive breast cancer.

## 1. Introduction

Neoadjuvant therapy is now the standard of care for most patients diagnosed with early HER2-positive breast cancer [1,2,3]. It is believed that, in the case of patients with a higher risk of recurrence, especially with confirmed axillary lymph node involvement and a lack of hormone receptors, the so-called dual anti-HER2 blockade with pertuzumab and trastuzumab in combination with chemotherapy is more effective than trastuzumab with chemotherapy [1,2,3]. Pertuzumab is a humanized monoclonal antibody that binds HER2 to a different epitope of the HER2 extracellular domain than trastuzumab, preventing HER2 dimerization with other ligand-activated HER2 receptors. The combination of pertuzumab, trastuzumab, and docetaxel improved pCR rates compared with trastuzumab and docetaxel therapies, from 21% to 39% [4,5]. In terms of survival, higher pCR rates are correlated with longer survival rates in this population [6,7].

Anthracyclines may be used sequentially, followed by taxanes in combination with anti-HER2 drugs, or an anthracycline-free regimen containing taxanes and carboplatin with targeted therapy is recommended [1,2,3]. The results of previous studies confirm the similar efficacy of both treatment modalities, with a lower risk of cardiotoxicity in the case of anthracycline-free regimens [8,9,10,11].

The response to neoadjuvant treatment is a source of important information about tumour biology and is a well-evaluated prognostic factor [7,12]. Based on the evaluation of the surgical specimens for pathologic complete response (pCR), decisions are made to continue the current therapy (trastuzumab +/− pertuzumab) following surgery or, if minimal residual disease is found, to switch to trastuzumab–emtansine to optimize long-term outcomes [13,14,15]. 

In recent years, the question of providing optimal treatment options for older women with breast cancer has been increasingly discussed. Available data on breast cancer rates in the U.S. demonstrate that most patients newly diagnosed with breast cancer are below the age of 65 (58%), whereas most breast cancer deaths occur in women aged ≥ 65 years (60%) [16]. 

In Poland, 18,869 women were diagnosed with breast cancer in 2018, of which 10,523 (66%) were below the age of 65. In the same year, there were 6895 deaths from breast cancer, of which 4609 (67%) were in women aged 65 and older [17].

Although many publications demonstrate that older women with no co-existing medical conditions tolerate toxic treatment regimens (such as TCbH-P or TCH) well, the percentage of older patients in randomized trials is small [18,19]. In fact, most authors emphasize that older women face an increased risk of severe treatment-induced toxicities, in particular of left ventricular systolic dysfunction (LVSD) [20,21]. Studies show that nearly 4% of patients develop congestive heart failure (CHF) during trastuzumab therapy. In patients aged over 60 years, this risk is increased to up to 5.4% [22].

### 1.1. Aim

The primary objective of this study was a retrospective evaluation of the efficacy and safety of the TCbH-P regimen (docetaxel, carboplatin, trastuzumab, and pertuzumab) in patients eligible for treatment with this neoadjuvant regimen in the Breast Cancer and Reconstructive Surgery Department. In addition, the data obtained were analysed to account for the age of the patients (<60 years old vs. ≥60 years old). The age limit was set at 60 years, as a similar age limit is usually set when patients are eligible for dose-neoadjuvant chemotherapy [2,3].

### 1.2. Ethics Statement

The study protocol was approved by the Ethics Committee of Maria Sklodowska-Curie National Research Institute of Oncology (No 12/2021). The study was performed per Good Clinical Practice standards and the ethical principles originating from the Declaration of Helsinki. All the patients provided informed consent for the use of their data for research purposes.

## 2. Materials and Methods

We analysed the medical records of breast cancer patients who were primarily treated with neoadjuvant therapy in the form of the TCbH-P regimen from 20 January 2018 to 10 December 2018 and then underwent surgery +/− radiotherapy. Radiotherapy was used in all patients who underwent tumourectomy and in patients after mastectomy with the T3 or T4 feature and with metastases to 4 or more axillary lymph nodes.

All patients met the following criteria: ECOG 0-1 performance status, histopathological diagnosis of HER2-positive invasive breast cancer, breast cancer staging (cT1-4, cN0-3, M0), neoadjuvant therapy with a TCbH-P regimen, and a baseline left ventricular ejection fraction (LVEF) of ≥50%.

Initially, a core needle biopsy was used to diagnose a breast tumour. Suspicious axillary lymph nodes were evaluated using an ultrasound (US)-guided fine needle biopsy. The presence of an oestrogen receptor (ER) and progesterone receptor (PR) was identified when ≥1% of nuclei were stained positive; if <1% of nuclei stained positive, it was considered a negative result. The HER2 status was considered positive based on an immunohistochemistry score of 3+ or 2+, which was confirmed with a positive FISH test [1].

All the patients received the TCbH-P regimen (docetaxel, carboplatin, trastuzumab, and pertuzumab) at the following doses: docetaxel, 75 mg/m^2^; carboplatin (AUC 6 mg/mL/min) intravenously, once every 3 weeks; trastuzumab, loading dose of 8 mg/kg followed by 6 mg/kg intravenously, once every 3 weeks; pertuzumab, loading dose of 840 mg followed by 420 mg intravenously, once every 3 weeks. 

All the patients underwent surgery after 6 cycles of neoadjuvant therapy. The response to treatment was assessed using the residual cancer burden (RCB) calculator (http://www3.mdanderson.org/app/medcalc/index.cfm?pagename=jsconvert3, accessed on 23 February 2022). Since T-DM1 (trastuzumab emtansine) is not currently reimbursed in Poland, trastuzumab (without pertuzumab, which is also not reimbursed in Poland for adjuvant therapy) was continued postoperatively for up to 18 cycles, whether the pCR was achieved or the minimal residual disease remained present.

All the patients with positive hormone receptors also received hormone therapy after surgery, as per the current guidelines, for at least 5 years.

Prior to the neoadjuvant therapy, the patients underwent mammography and were given breast and regional lymph nodes US, abdominal US, or computed tomography (CT), chest X-ray or CT, bone scintigraphy, ECG, echocardiography, and blood tests. 

All patients routinely received peg-GCSF after each course of chemotherapy.

Adverse events were assessed according to the Common Terminology Criteria for Adverse Events, Version 4.0 [23]. Echocardiography was used to monitor the left ventricular ejection fraction (LVEF); it was performed before the start of treatment and then every 12 weeks during the neoadjuvant therapy and every 3 months during adjuvant therapy.

All patients were evaluated for response every 6 weeks using breast and lymph node US.

### Statistical Analysis

Descriptive statistics were used to describe the characteristics of the study group: mean, median, first and third quartile (IQR) values, and range. The normality of the distribution of the individual parameters evaluated in the study was verified using the Shapiro–Wilk test. In the case of a normal distribution, Student’s t-test was used to compare the mean values of independent variables. For the other parameters without normal distributions, appropriate methods of statistical analysis were selected based on non-parametric tests. The Mann–Whitney U test was used to compare numerical variables between the two groups observed. Spearman’s rank correlation coefficient (monotonic relationships, linear or not) and the Pearson correlation coefficient (linear monotonic relationships) were used to examine the existence of monotonic relationships between two variables. All calculations and graphs were performed using the R stats package, Version 4.0.2.

## 3. Results

Thirty-four patients meeting the eligibility criteria were included in the study. Patient demographics are described in Table 1.

The assessment of the BRCA 1/2 mutation was performed in 12 patients with a burdened family history. No mutation was detected in any case.

The median age of the group was 46 (30–68) years old, of which 58.8% were patients diagnosed with luminal B, HER2-positive cancer. Only seven patients were ≥ 60 years (20.6%), due to the caution in qualifying older patients for a rather toxic treatment regimen, as there was a small number of research reports regarding the safety of treatment in this group of patients. Four patients in this group were diagnosed with breast cancer in the cT2 N0 stage; in two, the cT2 N1 stage was detected; and only one was treated in stage IIIC of breast cancer (cT4N2). 

No major medical burden was found in any of the patients. Before they were started on chemotherapy, all patients had a normal LVEF > 50%. 

The size of the breast tumour during therapy decreased, which was evaluated clinically and by US.

A total of 58.8% of the patients underwent mastectomy (+immediate breast reconstruction in eight patients) and 38.3% underwent wide local tumour excision; 1 patient (2.9%) did not undergo breast surgery as there was no tumour in the breast (T0). A total of 53% of patients underwent axillary lymphadenectomy, while 47.1% of patients had a sentinel node (SN) procedure (in 8.9% of the patients, targeted axillary dissection was performed).

The most common severe complications (grade 3 or 4) were neutropenia and febrile neutropenia. Among patients aged ≥ 60 years, the most common adverse reactions of grade 3/4 were diarrhoea (28%), neutropenia (28%), and febrile neutropenia (28%).

Treatment toxicities are shown in Table 2.

Ten patients (29.4%) had their dose of carboplatin reduced, and four patients (11.7%) had their dose of docetaxel reduced.

Sixteen patients received ESA supportive therapy (at least one dose of darbepoetin) during chemotherapy. Two patients required a transfusion of red blood cells due to grade 3/4 anaemia. 

All patients completed the expected six cycles of treatment and underwent surgery.

None of the patients had a decrease in LVEF or clinical manifestations of heart failure during the entire period of systemic therapy. No complication-related mortality was observed.

Pathologic complete response was observed in 18 (52.9%) patients. The average time from disease diagnosis to last follow-up was 27.15 (+/−5.53) months. During this time, relapse was observed in one (2.9%) patient (68-year-old female patient with HER2-positive luminal cancer, cT2 N0).

The characteristics of the responses obtained are shown in Table 3.

A statistically significant relationship was identified between the type of response achieved (pCR vs. non-pCR) and the presence of ER (*p* < 0.05). In a univariate analysis, the odds of achieving pCR were 14 times higher in the ER-negative arm compared with the ER-positive group (*p* = 0.004). This was confirmed by the multivariate analysis: ER status (OR: 0.01, 95% CI: 0.00–0.16; *p* = 0.005).

No correlation was identified between the occurrence of pCR and the primary stage of the disease.

The results describing the response to treatment depending on age, disease staging, and ER and PR status are shown in Table 4.

**Figure 1:**
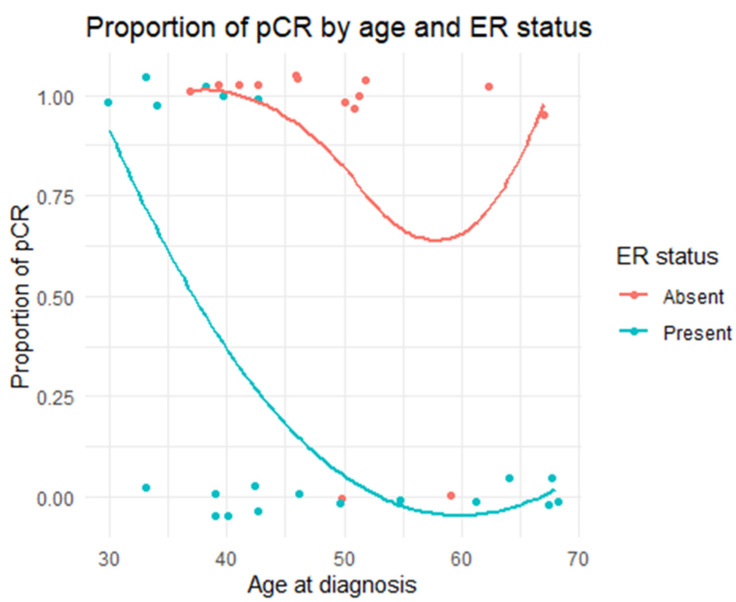
Relationship between achieving a pathologic complete response, the ER status, and patient age.

It was observed that in the group of patients with non-luminal HER2-positive cancer, the pCR rate did not depend on the age of patients, compared with patients with luminal HER2-positive cancer, of whom mainly young patients achieved pCR (Figure 1). As such, the use of aggressive chemotherapy in combination with dual anti-HER2 blockade (pertuzumab + trastuzumab) in patients over 60 years old diagnosed with luminal HER2-positive cancer might not be necessary due to the small chance of achieving the expected pCR. The use of trastuzumab with weekly paclitaxel is probably a sufficient method of treatment in this group of patients. However, it must be emphasized that this hypothesis cannot be definitively proven due to the small number of subjects. 

## 4. Discussion

The diagnosis of breast cancer with high HER2 expression levels is associated with a worse prognosis and more dynamic disease progression. Significant improvements in therapy outcomes for this biologic subtype were achieved when HER2 blockers were introduced into routine management [24,25]. 

Improved long-term outcomes were also achieved when neoadjuvant treatment with anti-HER2 therapy was included in the standard of care [4,5,6,7,8,9]. The use of neoadjuvant therapy can facilitate surgical treatment, increase the number of patients eligible for breast-conserving therapy (BCT), determine the degree of sensitivity to the therapy used, and identify patients who do not achieve pCR and thus should be treated more intensively after surgery [1,2,3,13]. It is currently recommended that patients with HER2-positive cancer at high risk of recurrence or with HER2-positive locally advanced cancer should be treated with a combination of chemotherapy and dual anti-HER blockade, i.e., trastuzumab and pertuzumab [4,5,26]. The most effective regimens for neoadjuvant treatment are considered to be an anthracycline-free chemotherapy regimen, i.e., TCbH-P, and anthracycline-containing sequential regimens, i.e., AC-THP. It should be emphasized that anthracycline-containing regimens are associated with a higher risk of cardiotoxicity [8,27].

The TRYPHAENA and TRAIN-2 studies did not demonstrate a significant difference in pCR rates between regimens with or without anthracyclines [8,27], with pCR rates exceeding 65% in patients receiving anthracycline-free regimens. In this context, the TCbH-P regimen seems to be a more rational option for neoadjuvant therapy, in particular since most women undergoing the treatment are likely to survive for many years without cancer recurrence [28,29,30,31]. 

Several clinical trials have evaluated the efficacy and safety of the TCbH-P regimen as a perioperative treatment [5,8,11]. In most publications, the percentage of pCR was 55–66% [8,9,27], whereas in our study we identified pCR in only 52.9% of women. We believe this is due to the high proportion of HER2-positive luminal cancer patients, with pCR found in only 33.3% (vs. 66.7% in ER-negative, HER2-positive patients), and the significantly lower chance of pCR observed in our patients aged 60 years and older, compared with younger patients (11.1% vs. 89.9%).

In observational studies describing everyday clinical practice, younger patients with no significant co-existing medical conditions are often selected for treatment with dual-blockade anti-HER2 regimens, TCbH-P in particular [32,33]. The results of our study support the validity of such choices since, especially for ER-positive patients, the benefit of using a relatively toxic TCbH-P regimen is unlikely to carry any extra benefits and may lead to serious complications.

The most common adverse events found in our study were anaemia, weakness, neuropathy, and neutropenia. Diarrhoea was observed in our patients less frequently (26%) than in other studies, whereas most publications report the occurrence of diarrhoea in 40–56% of patients started on the treatment [8,9,27,32,33]. It should be noted, then, that we observed diarrhoea significantly more often in patients aged 60 years and older (71%) than in younger patients. 

We reported thrombocytopenia slightly less frequently than in other studies (38% of patients only), with no patient having grade 4 thrombocytopenia, which is probably related to the relatively high number of patients that received a reduced dose of carboplatin (29.4%). The reasons for the dose reduction were mainly neuropathy, neutropenia, and anaemia.

We did not observe symptomatic heart failure or a significant decrease in LVEF (less than 10% compared with baseline) in any of the assessed patients.

The disadvantages of our study are the small number of subjects and the relatively short follow-up time (slightly more than 2 years); nevertheless, it represents a group treated in one centre with the same eligibility criteria and supportive treatment applied during therapy. It should also be emphasized that we analysed the effectiveness and toxicity of the treatment taking into account the age of the patients. Grade 3 and 4 toxicity was more common in patients over 60 years of age, and the achieved pCR rate was only 11.1% in patients with the HER2-positive subtype of unclear breast cancer. In most of the publications available, the TCbH-P program is not used in patients over 65 years of age, and often in patients over 60 years of age [9,10,11,32,33]. Our observations confirm that in the group of elderly patients, the use of an aggressive treatment regimen is not associated with the expected response.

## 5. Conclusions

Our study confirmed that the TCbH-P regimen is safe and relatively effective in the neoadjuvant treatment of patients with HER2-positive breast cancer. No case of myocardial dysfunction or significant decreases in LVEF were observed. However, our results suggest that consideration should be given to whether the TCbH-p regimen is the optimal choice for luminal B HER2-positive cancer patients over the age of 60. In this group, the chance of achieving pCR, based on our assessment, is low, while serious toxic effects are a risk during treatment. Therefore, we suggest the need for further research in this direction.

## Figures and Tables

**Table 1:** Patient characteristics.

Variable	Parameter	N = 34	%
Age at diagnosis of breast cancer (years)	Median (IQR)	46 (39.25–54.25)	
	Range	30–68	
Age at diagnosis of breast cancer (years)—breakdown	<60 years old	27	79.4%
	≥60 years old	7	20.6%
cT	0	1	2.9%
	1	3	8.8%
	2	21	61.8%
	3	6	17.6%
	4d	3	8.8%
cN	0	13	38.2%
	1	14	41.2%
	2	5	14.7%
	3	2	5.9%
ER	Positive	20	58.8%
	Negative	14	41.2%
PR	Positive	15	44.1%
	Negative	19	55.9%
Type of treatment—breast	Mastectomy	12	35.3%
	Mastectomy + immediate breast reconstruction	8	23.5%
	Breast-conserving surgery (BCS)	13	38.3%
	No treatment (T0 patient)	1	2.9%
Type of treatment—axilla	Axillary dissection	18	53%
	Sentinel node (SN)	13	38.2%
	Targeted axillary dissection (TAD)	3	8.9%

**Table 2:** Treatment toxicity.

Toxicity	Incidence (Any Grade)			Grade 3 or 4		
	Any	Patients < 60	Patients ≥ 60	Any	Patients < 60	Patients ≥ 60
Diarrhoea	9 (26%)	4 (15%)	5 (71%)	2 (6%)	0	2 (28%)
Thrombocytopenia	13 (38%)	9 (33%)	4 (57%)	0	0	0
Neutropenia	14 (41%)	10 (37%)	4 (57%)	4 (12%)	2 (7%)	2 (28%)
Febrile neutropenia	4 (12%)	2 (7%)	2 (28%)	4 (12%)	2 (7%)	2 (28%)
Anaemia	27 (79%)	23 (85%)	4 (57%)	2 (6%)	1 (3.5%)	1 (14%)
Fatigue	24 (70%)	19 (70%)	5 (71%)	2 (6%)	1 (3.5%)	1 (14%)
Neuropathy	22 (65%)	17 (63%)	5 (71%)	0	0	0
Mucositis	3 (9%)	2 (7%)	1 (14%)	0	0	0
Cardiac dysfunction	0	0	0	0	0	0

**Table 3:** Characteristics of achieved responses to treatment.

Characteristic	Parameter	% (N = 34)
Achieved response to neoadjuvant treatment	pCR	52.9% (N = 18)
	non pCR	47.1% (N = 16)
Residual cancer burden (RCB)—only patients with non-pCR	I	20.6% (N = 7)
	II	11.8% (N = 4)
	III	14.7% (N = 5)

**Table 4:** Response to treatment depending on age, disease stage, and ER and PR status.

Variable	Parameter	pCR (N = 18)	Non-pCR (N = 16)	*p*-Value
Age at diagnosis of breast cancer (years)	N	18	16	0.07512
	Median	43	50	
	Range	30–67	33–68	
Age at diagnosis of breast cancer (years)—breakdown	<60 years old	88.9% (N = 16)	68.8% (N = 11)	0.2143
	≥60 years old	11.1% (N = 2)	31.2% (N = 5)	
cT	0	5.6% (N = 1)	0% (N = 0)	0.8128
	1	11.1% (N = 2)	6.2% (N = 1)	
	2	61.1% (N = 11)	62.5% (N = 10)	
	3	11.1% (N = 2)	25% (N = 4)	
	4d	11.1% (N = 2)	6.2% (N = 1)	
cN	0	38.9% (N = 7)	37.5% (N = 6)	1
	1	38.9% (N = 7)	43.8% (N = 7)	
	2	16.7% (N = 3)	12.5% (N = 2)	
	3	5.6% (N = 1)	6.2% (N = 1)	
ER	Positive	33.3% (N = 6)	87.5% (N = 14)	0.0019
	Negative	66.7% (N = 12)	12.5% (N = 2)	
PR	Positive	27.8% (N = 5)	62.5% (N = 10)	0.0824
	Negative	72.2% (N = 13)	37.5% (N = 6)	

## Data Availability

The data presented in this study are available on request from the corresponding author.
